# Three-Dimensional Convolutional Neural Network-Based Prediction of Epidermal Growth Factor Receptor Expression Status in Patients With Non-Small Cell Lung Cancer

**DOI:** 10.3389/fonc.2022.772770

**Published:** 2022-02-02

**Authors:** Xuemei Huang, Yingli Sun, Mingyu Tan, Weiling Ma, Pan Gao, Lin Qi, Jinjuan Lu, Yuling Yang, Kun Wang, Wufei Chen, Liang Jin, Kaiming Kuang, Shaofeng Duan, Ming Li

**Affiliations:** ^1^Department of Radiology, Huadong Hospital Affiliated With Fudan University, Shanghai, China; ^2^Dianei Technology, Shanghai, China; ^3^Precision Health Institution, GE Healthcare, Shanghai, China

**Keywords:** NSCLC, EGFR, tomography, radiogenomics, deep learning, machine learning

## Abstract

**Objectives:**

EGFR testing is a mandatory step before targeted therapy for non-small cell lung cancer patients. Combining some quantifiable features to establish a predictive model of EGFR expression status, break the limitations of tissue biopsy.

**Materials and Methods:**

We retrospectively analyzed 1074 patients of non-small cell lung cancer with complete reports of *EGFR* gene testing. Then manually segmented VOI, captured the clinicopathological features, analyzed traditional radiology features, and extracted radiomic, and deep learning features. The cases were randomly divided into training and test set. We carried out feature screening; then applied the light GBM algorithm, Resnet-101 algorithm, logistic regression to develop sole models, and fused models to predict EGFR mutation conditions. The efficiency of models was evaluated by ROC and PRC curves.

**Results:**

We successfully established Model_clinical_, Model_radiomic_, Model_CNN_ (based on clinical-radiology, radiomic and deep learning features respectively), Model_radiomic+clinical_ (combining clinical-radiology and radiomic features), and Model_CNN+radiomic+clinical_ (combining clinical-radiology, radiomic, and deep learning features). Among the prediction models, Model_CNN+radiomic+clinical_ showed the highest performance, followed by Model_CNN_, and then Model_radiomic+clinical_. All three models were able to accurately predict EGFR mutation with AUC values of 0.751, 0.738, and 0.684, respectively. There was no significant difference in the AUC values between Model_CNN+radiomic+clinical_ and Model_CNN_. Further analysis showed that Model_CNN+radiomic+clinical_ effectively improved the efficacy of Model_radiomic+clinical_ and showed better efficacy than Model_CNN_. The inclusion of clinical-radiology features did not effectively improve the efficacy of Model_radiomic_.

**Conclusions:**

Either deep learning or radiomic signature-based models can provide a fairly accurate non-invasive prediction of EGFR expression status. The model combined both features effectively enhanced the performance of radiomic models and provided marginal enhancement to deep learning models. Collectively, fusion models offer a novel and more reliable way of providing the efficacy of currently developed prediction models, and have far-reaching potential for the optimization of noninvasive EGFR mutation status prediction methods.

## Introduction

Lung cancer is the leading cause of cancer-related deaths, with incidence and mortality rates of approximately 11.4% and 18%, respectively, and is the second-highest incidence rate in the world (1). Non-small cell lung cancer is the main pathological form and accounts for approximately 80-90% of all lung cancers (2). Targeted therapy has become one of the first-line standard treatments for non-small cell lung cancer patients; because this form of treatment can effectively improve their prognosis, prolong the PFS and OS, compared with traditional means of treatment, like chemotherapy (3–6). In patients with non-small cell lung cancer, EGFR is responsible for approximately 10-20% of all and is the most predominant driver mutations target for targeted therapy (7). As a consequence, EGFR-TKI therapy plays a pivotal role in the targeted therapy of patients with non-small cell lung cancer.

Prior to EGFR-TKI treatment, it is essential to perform EGFR genetic testing to clarify the presence of EGFR mutations. There are several methods that can be used to detect EGFR mutations, including tissue biopsy, liquid biopsy, and radiogenomics.

Histopathological biopsy has been the gold standard in terms of high sensitivity and specificity in clinical disease and genetic diagnosis. However, it still has the following restrictions: 1. High sample size threshold, requiring at least 20% of tumor cells in the sample to be detectable (8). 2. As the tumor genotype itself possesses heterogeneity (9–11), while part of the samples are taken from puncture biopsies, so there is a risk of sampling bias, which means that the gene mutation status detection result may not correspond to the authentic condition and is not representative of the whole gene expression profile of the cancer spot. 3. Because of heterogeneity of neoplastic cell genetic status, disease progression or drug resistance commonly occurs in terminal period of the disease, so that re-biopsy is necessitated to evaluate the disease and clarify if a drug resistant mutation such as T790M (12) has evolved to instruct subsequent treatment, yet the biopsy is an invasive operation with complications including pneumothorax and bleeding, and often not feasible due to the patient’s physiological issues in terminal course of the disease, thus blocking the personalized health maintenance strategy. 4. More expensive, with higher standards of material storage and instrumentation, which is not conducive to applying and promoting in certain impoverished and remote areas.

Liquid biopsy refers to the extraction of tumor gene-carrying agents from body fluids, such as Circulating tumor-derived DNA, cell-free DNA, etc., for detecting the relevant genetic alterations, and it has the merits of real-time detection and minor invasion, however, due to the existence of tumor spatial heterogeneity, it may not be capable of accurate localization or representing the true mutation level in the whole tumor; besides, in the early stage, there are often no circulating tumor cells in body fluids, and their concentration is susceptible to influence, resulting in an insufficient sample size. Presently, cell-free DNA is the only liquid biopsy marker recommended for insufficient volume of pathology biopsies or to monitor the presence of EGFR T790M mutations with disease progression or drug resistance (13, 14). Moreover, a recent study (15) indicates that the sensitivity and specificity of this technique are poor and that the practical use of this method remains controversial.

Hence enabling a holistic and comprehensive analysis of the lesion by surmounting the obstacle posed by genetic heterogeneity is now a much desirable claim.

Regarding the aforementioned downsides of tissue biopsy pathology and liquid biopsy, researchers have exploited the advancing artificial intelligence to provide a technology with promising clinical applicability - radiogenomics (16–19). It is a group of imaging biomarkers that can offset the constraints of tissue biopsies and liquid biopsies by effectively and non-invasively projecting the mutational status of genes such as EGFR and ALK *via* artificial intelligence methodology, enabling high-throughput molecular biological information, as tumor heterogeneity and genotype, which is not visible to the naked eye, and converting them into digital signals (deep learning features or radiomics features), quantifying and characterizing them to facilitate disease diagnosis as well as monitoring and guiding targeted therapy decision-making. Several researchers have reported that radiogenomics represents a promising application for EGFR gene detection. Both deep learning models (20, 21) and radiomic models (22–24) have been shown to be more precise in predicting the mutational status of EGFR. However, most studies have applied deep learning and radiomic features in an independent manner; far fewer studies have attempted to combine these two features. A previous study reported the successful creation of an EGFR mutation prediction model based on the fusion of these two features (25). However, this study only included patients with solid lung adenocarcinoma. Furthermore, some of the images used were thick; this may have led to the loss of valuable features. Moreover, the EGFR mutation sites described in this previous study only contained exons 19 and 21. This is a concern because the ground-glass component within a cancer site maybe can provide more heterogeneous information than the solid component (26).

In the present study, we aimed to investigate and validate whether a prediction model incorporating deep learning features and radiomic features can improve the performance of the current mainstream models for the non-invasive prediction of EGFR mutations. To expand the application of radiomic features and deep learning features for non-invasive gene detection, we recruited a large number of patients with ground glass non-small cell lung cancers and used thin-layer images to avoid or minimize the loss of effective features.

## Materials and Methods

**Figure 1:**
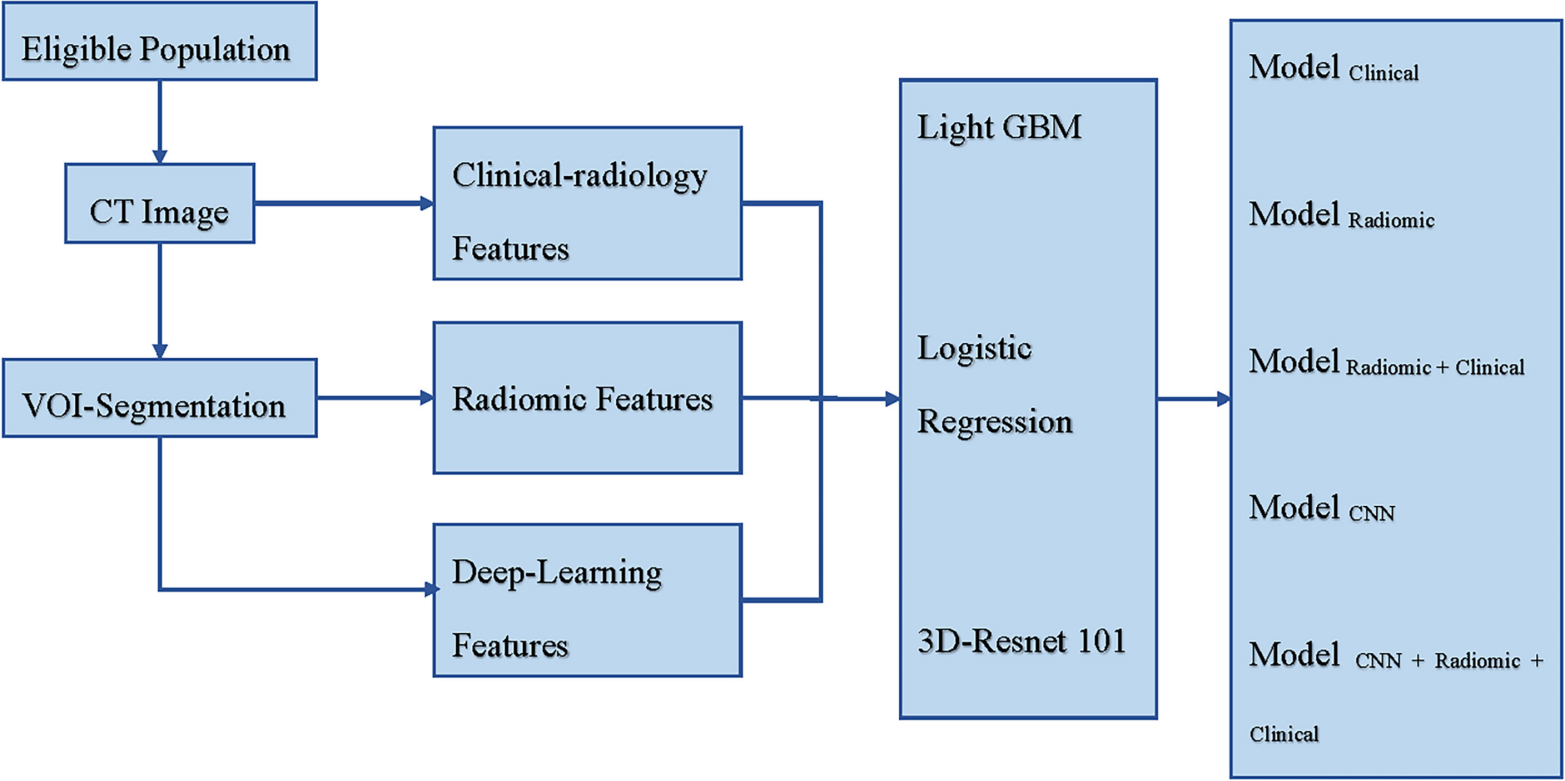
Schematic for the models’ construction. CT, Computed Tomography; VOI, Volume Region of Interest; Light GBM, Light Gradient Boosting Machine; Res-Net, Residual Network; Modelclinical incorporated clinical-radiology features, Modelradiomic incorporated radiomic features, Modelradiomic+clinical combined clinical-radiology and radiomic features, ModelCNN incorporated deep learning features, and ModelCNN+radiomic+clinical combined clinical-radiology, radiomic, and deep learning features.

**Figure 1** shows a schematic for how the models were constructed.

### Population and Clinicopathological Data

Before initiating the research, we derived the AUC value of the radiogenomic model from that of several previous studies, which was about 0.70-0.95, and made a sample size estimation based on this data, which resulted in a predicted maximum number of 104 people needed. Later, after reminded by deep learning experts, and given the demand for large data samples for deep learning, it was decided to extend the sample on the pre-estimated sample size. We ultimately retrospectively recruited patients with pathologically confirmed primary non-small cell lung cancer between 4^th^ June 2019 and 21^st^ January 2021 at the Huadong Hospital, Fudan University, Shanghai, China. All patients were screened according to strict inclusion and exclusion criteria; this process led to the inclusion of 1074 eligible patients. The inclusion criteria were as follows: (1) detailed EGFR gene test reports were available, (2) the interval between chest CT examination and surgery was within 1 month, and (3) pathological samples were obtained from surgically resected specimens. The exclusion criteria were as follows: (1) image layer thickness greater than 1.5 mm, (2) images with severe motion artifacts or conditions such as pleural effusion or obstructive pneumonia that may affect detailed observation, (3) preoperative history of tumors or a history of lung surgery, and (4) an inability to convert image format or extract features for unknown reasons. For each patient, we collated a complete range of clinicopathological data, including age, gender, smoking history, invasive degree, and EGFR mutation status. The basic principle of the training/test split is to maintain a general fraction of positive samples in each subset. We used the train_ test_ split function in Scitkit-learn 0.24.2 to perform a random selection of training/test data while maintaining roughly the same proportion of positives/negatives in both subsets, and to guarantee reproducibility, we kept the seed of the random number generator fixed at 42, which is a prevalent alternative among deep learning researchers. All cases were randomly divided into a training set (770 cases) and a test set (304 cases).

### CT Instrument and Parameters

All patients were scanned with a GE Discovery CT750HD or LightSpeed VCT or Somatom Sensation 16 CT system, operating with the following parameters: tube voltage: 120 kV; tube current: 200 mA; reconstruction algorithm: STND/medium sharp; and layer thickness: 1.00/1.25/1.5 mm. Three apparatus distribution for Discovery: VCT: Somatom (training set-340:184:246; test set-135:83:86) The scan phase was set to the deep inspiratory phase and the patient was scanned in the supine position. Images were acquired in the DICOM format. Further details of the parameters used for CT are shown in **Supplementary Table 1**.

### Histopathology and the Diagnosis of EGFR Status

The histopathological type of non-small cell lung cancer was identified by our diagnostic pathologists for secondary diagnosis using the 2011 international and multidisciplinary classification guidelines proposed by the International Association for the Study of Lung Cancer/American Thoracic Society/European Respiratory Society (27) and the World Health Organization (WHO) 2015 guidelines for lung cancer classification (28). The mutation status of EGFR exons 18, 19, 20 and 21 (which are associated with drug targets) was detected using a real-time fluorescent PCR-based amplification refractory mutation system and a human EGFR gene mutation real-time reverse transcription-polymerase chain reaction diagnostic kit (AmoyDx, Xiamen, China).

### VOI Segmentation and Radiology Features

First, the pixels in the raw DICOM images were uniformly transformed to a layer thickness of 1 mm. Then, the VOI of the cancer was manually segmented by a junior diagnostician (Reader 1) using the open-source software 3D-slicer (https://www.slicer.org/) ensuring that large blood vessels and fibrous connective tissue was avoided during contouring. A secondary manual correction was performed by a senior physician (Reader 2). Another senior diagnostician (Reader 3) analyzed and recorded the CT radiology features of the tumor while remaining blinded to the EGFR mutation status and pathological subtypes. Reader 3 recorded a range of data, including location, cancer density, border, vacuole sign, air bronchogram sign, spiculation sign, lobulation sign, halo sign, vascular alteration, pleural indentation, and umbilicated indentation. In case of disagreement, a second evaluation was performed by another senior diagnostician (Reader 4); the results were recorded after discussion and agreement. All images were observed with a window position of -500 HU and a window width of 1500 HU. In the following features description, for the sake of brevity, we merge the radiology features with the clinical features, and use the description of the clinical features uniformly.

### Analysis of Radiomic Features

The outlined VOIs were placed into Pyradiomics (29) (version 3.0 software) to extract radiomic features. Pyradiomics is an open-source python package for extracting radiomic features from medical imaging.

### Reproducibility Analysis

To assess the reproducibility and stability of the radiomic features, 60 patients were randomly selected by the diagnostician (Reader 1) for secondary manual segmentation of the tumor VOI after one month. The radiomic features were extracted and subjected to ICC analysis; features with an ICC index≥0.95 were selected for subsequent model construction.

### Clinical and Radiomic Models

To further identify redundant features and improve the performance of the radiomic model, we re-screened the initial radiomic features by considering mutual information between each feature and the mutation status of the EGFR gene. The mutual information between two random variables is a non-negative value that measures the dependence between the two variables (30). This function relies on a non-parametric approach based on entropy estimation from K-nearest neighbor distances and can be used for the univariate selection of features. Ultimately, we filtered the top 10% of features with the highest mutual information in the training set to develop the model. Then, we retained the same 10% of features in the test set to evaluate model performance. Based on the screened radiomic features and clinical features, we established Model_radiomic_ and a fusion model (Model_radiomic+clinical_) using the Light GBM algorithm (31). To avoid overfitting, during model construction, we adjusted several hyperparameters, including learning rate, data down-sampling ratio, feature down-sampling ratio, and L1/L2 regularization strength. The learning rate was tuned before the steady convergence of the training and validation losses of the model was observed. Intensity of overfitting prevention enhances when we decrease the data down-sampling rate, feature down-sampling rate, or augment the L1/L2 regularization strength.

### Deep Learning Model

Both the original CT images and the mask of the VOI were resampled to a space-occupying 1 mm × 1 mm × 1 mm. Next, we counted the spanning distribution of the cancer in three dimensions, and selected 64 mm × 64 mm × 64 mm as the input size for deep learning to ensure that the cropped input size could cover the extent of all lung nodules. The HU values of this patch were processed using the clip of the lung window [(-1000, 400)] and subjected to the minimum-maximum normalization process. Next, the resultant data were imported into the Ampyx 3D ResNet101 network to facilitate the creation of Model_CNN_, a model that featured only deep learning features. 3D ResNet101 (32) is a well-characterized and broad applicable neural network in the field of deep learning, and remains considered as a strong comparative baseline in computational vision research. Compared to its successor, its network is relatively simplistic, which further alleviates overfitting and thus enables a more robust model ultimately. The model was optimized with AdamW (33) with a maximum learning rate of 0.001. We also used a cosine annealing schedule (34) to gradually reduce the model to 10^-6^ within 500 epochs. To further suppress overfitting and enhance the robustness of the model, we performed data augmentation using random rotation, random flip, and mix-up (35) with an α of 0.2. Since the objective of this study was not to innovate new neural network structures, the hyperparameters of this ResNet101 model were adjusted following the configuration given in the Torch Vision Python package.

### Fusion of Clinical-Radiomic-Deep Learning Features Model

Since the deep learning features and clinical/radiomic features are totally different in terms of both data distribution and expressed meaning, and the number of filtered clinical/radiomic features is larger than that of deep learning features, the weight of clinical/radiomic features tends to be greater if the features are simply combined, and the model performance is poor. Therefore, we finally opted to model the prediction probability of Model_CNN_ and that of Model_radiomic+clinical_, and constructed a metamodel Model_CNN+radiomic+clinical_ using logistic regression. Essentially, we perform 5-fold cross-validation on the Model_CNN_ and the Model_radiomic+clinical_ respectively in the training set, and build a logistic regression Model_CNN+radiomic+clinical_ by weighting the probabilities calculated from the two models.

### Model Evaluation

Next, the ROC curve, AUC value, and PRC curve were used to evaluate the predictive performance of each model. To verify whether the fusion model performs better than the sole model and whether the improvement in model performance is statistically significant, the De-long test is applied to compare the performance variation of each model.

### Statistical Analysis

This research was carried out with Python (version 3.8.10). Modeling of radiomics features, clinical features, and the concatenation of both was done using Light GBM (version3.2.1). CNN experiments were conducted using PyTorch (version1.8.1). The logistic regression model fusing clinical, radiomic, and deep learning features were provided by Scitkit-Learn (version0.24.2.). DeLong tests were done in MedCalc (version20.0009). The sample size was calculated in PASS 15 (Power: 0.90; Alpha: 0.05; AUC1:0.7; Two-Sided).Univariate analysis and multivariate logistic analysis using SPSS (version23.0). The normality distribution of the continuous variables was verified with the Kolmogorov-Smirnov test(P<0.001). Continuous variables were analyzed using Mann-Whitney U test. Categorical variables were analyzed using chi-square tests or Fisher’s exact test. p-values less than 0.05 were considered statistically significant.

## Results

A total of 1074 eligible non-small cell lung cancer cases were enrolled in this study, including 527 wild-type EGFR cases and 547 EGFR mutant cases; there were 443 males and 631 females. Analysis of between-group discrepancy showed that there was no significant difference in the clinical-radiology characteristics when compared between the training and test sets, as detailed in **Supplementary Table 2**. The distribution of the clinical-radiology characteristics of EGFR mutant-type and wild-type cases within the training set is shown in **Table 1**. Screening of the training set revealed that six items (gender, age, invasive degree, cancer density, vacuole sign, and smoking history) were all independent predictors for EGFR mutation. Detailed statistics of clinical characteristics are shown in **Table 2**. In contrast, location, border, air bronchogram sign, spiculation sign, lobulation sign, halo sign, vascular alteration, pleural indentation, and umbilicated indentation, could not specifically identify EGFR mutation. For each case, 1218 radiomic features were extracted from the VOI; ICC analysis yielded a mean correlation coefficient of 0.96 ± 0.07. Subsequently, 243 radiomic features with coefficients <0.95 were excluded, and the top 10% of the radiomic features with the highest mutual information were identified, and used to build the model. Finally, six clinical features and 108 radiomic features were used to build the predictive models. The top 20 radiomic features selected are shown in **Supplementary Table 3**.

**Table 1:** The distribution of clinical-radiology features for EGFR mutant and wild type cases in the training set.

Characteristics	EGFR wild	EGFR mutation	*p-value*
**Gender**			0.004*
Male	176 (46.6)	142 (36.2)	
Female	202 (53.4)	250 (63.8)	
**Age**	57.5 (18.0)	60.0 (16.0)	0.004*
**Invasive Degree**			<0.001*
Non-invasive	64 (16.9)	27 (6.9)	
Micro-invasive	158 (41.8)	131 (33.4)	
Invasive	156 (41.3)	234 (59.7)	
**Location**			***0.248***
RUL	137 (36.2)	145 (37.0)	
RML	23 (6.1)	39 (9.9)	
RLL	64 (16.9)	59 (15.1)	
LUL	98 (25.9)	103 (26.3)	
LLL	56 (14.8)	46 (11.7)	
**Cancer density**			<0.001*
Pure GGO	72 (19.0)	29 (7.4)	
Mixed GGO	221 (58.5)	313 (79.8)	
Solid	85 (22.5)	50 (12.8)	
**Border**			***0.121***
Well-define	271 (71.7)	254 (64.8)	
Less-define	61 (16.1)	79 (20.2)	
Ill-define	46 (12.2)	59 (15.1)	
**Vacuolation**			<0.001*
Present	112 (29.6)	202 (51.5)	
Absent	266 (70.4)	190 (48.5)	
**Air Bronchogram**			<0.001*
Present	120 (31.7)	194 (49.5)	
Absent	258 (68.3)	198 (50.5)	
**Spiculation**			0.025*
Short	77 (20.4)	99 (25.3)	
Deep	26 (6.9)	32 (8.2)	
Mixed	60 (15.9)	81 (20.7)	
Absent	215 (56.9)	180 (45.9)	
**Lobulation**			***0.133***
Shallow	125 (33.1)	114 (29.1)	
Deep	4 (1.1)	11 (2.8)	
Mixed	248 (65.6)	263 (67.1)	
Absent	1 (0.3)	4 (1.0)	
**Halo**			0.006*
Present	46 (12.2)	76 (19.4)	
Absent	332 (87.8)	316 (80.6)	
**Vascular- Alteration**			***0.675***
Present	191 (50.5)	204 (52.0)	
Absent	187 (49.5)	188 (48.0)	
**Pleural- Indentation**			<0.001*
Present	133 (35.2)	187 (47.7)	
Absent	245 (64.8)	205 (52.3)	
**Umbilicated- Indentation**			0.001*
Present	29 (7.7)	59 (15.1)	
Absent	349 (92.3)	333 (84.9)	
**Smoke History**			0.020*
Yes	172 (45.5)	146 (37.2)	
No	206 (54.5)	246 (62.8)	

RUL, right upper lobe; RML, right middle lobe; RLL, right lower lobe; LUL, left upper lobe; LLL, left lower lobe; GGO, ground glass opacity; Categorical variables (e.g. gender) are expressed by a number (percentage), continuous variables (e.g. age) are expressed by the Median (interquartile range). *p<0.05 (significant), P-values taken with three decimal places equal to 0.000 are expressed as <0.001. The bolded values in the left column refer to the clinical-radiological features included in the statistical analysis of this study, and the bolded values in the right column refer to the P values, with P less than 0.05 as the criterion to evaluate whether they are statistically significant and whether they are included in the subsequent statistical sub-analysis. The data are bolded for the purpose of making them more prominent and clear only.

**Table 2:** | Statistical analysis outcome of clinical-radiology characteristics.

Selected Features	Univariate Analysis		Multivariate Analysis	
	Z or χ2	*P*	Regression coefficient	*P*
**Gender**	8.481	*0.004*	-0.649	***<0.001***
**Age**	-2.826	*0.004*	0.015	***0.037***
**Invasive Degree**	32.923	*<0.001*	-1.158	***<0.001***
**Cancer density**	42.991	*<0.001*	1.510	***<0.001***
**Vacuolation**	38.221	*<0.001*	0.571	***0.001***
**Air-Bronchogram**	25.088	*<0.001*	0.251	*0.165*
**Spiculation**	9.348	*0.025*	0.313	*0.253*
**Halo**	7.520	*0.006*	-0.506	*0.051*
**Pleural- Indentation**	12.418	*<0.001*	0.145	*0.493*
**Umbilicated- Indentation**	10.352	*0.001*	0.481	*0.093*
**Smoke History**	5.413	*0.020*	-0.335	***0.038***

Univariate Analysis: Continuous variables were analyzed using Mann-Whitney U test. Categorical variables were analyzed using chi-square tests or Fisher's exact test.

Features with bolded numbers of the P-value column are independent predictors.

**Figure 2:**
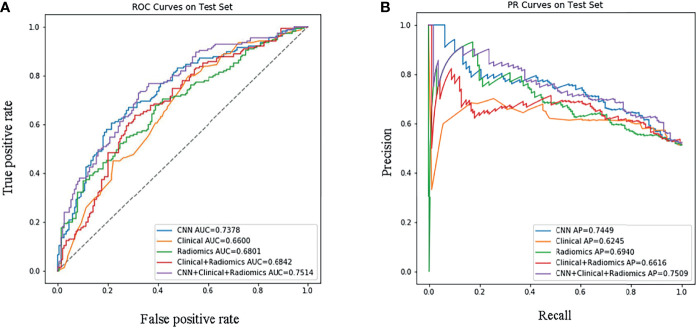
Performance evaluation of the models in the test set. **(A)** Receiver Operating Characteristic curve; **(B)** Precision-Recall curve. ‘CNN+Clinical+Radiomic’ refers to ModelCNN+radiomic+clinical, ‘Clinical+Radiomic’ refers to Modelradiomic+clinical, ‘Radiomic’ refers to Modelradiomic, ‘Clinical’ refers to Modelclinical, and ‘CNN’ refers to ModelCNN.

Next, we successfully built five prediction models: Model_CNN+radiomic+clinical_, Model_CNN_, Model_radiomic+clinical_, Model_radiomic_, and Model_clinical_. The performance of each model was verified in the test set, as shown in **Figure 2**. In the test set, the most effective prediction model, as based on the ROC curve, was Model_CNN+radiomic+clinical_ with an AUC of 0.751; this was followed by Model_CNN_, Model_radiomic+clinical_, and finally Model_clinical_. Our analysis showed that deep learning models and radiomic models both can predict EGFR mutations with the best levels of accuracy. Model_CNN+radiomic+clinical_, which featured both deep learning and radiomic features, showed more effective improvement than the mainstream radiomic models (Model_radiomic+clinical_ and Model_radiomic_), with p-values of 0.0067 and 0.0063, respectively. Although the Delong Test revealed that the difference in efficacy between the two models was not statistically significant, detailed analysis of the ROC and PRC curves showed that the fusion model (Model_CNN+radiomic+clinical_) was slightly more effective than the deep learning model (Model_CNN_). The Delong Test also showed that the difference in efficacy between Model_radiomic+clinical_ and Model_radiomic_ was also not statistically significant, and that the addition of clinical information did not enhance the efficacy of Model_radiomic_ (p = 0.876).

## Discussion

In this study, we developed a fusion model for predicting EGFR mutation levels in 1074 patients with non-small cell lung cancer by analyzing the clinical, radiology, radiomic, and deep learning features. The value of the combined model (Model_CNN+radiomic+clinical_) was more efficient than models based on radiomic or deep learning features alone, particularly those based on radiomic features. The general objectives of this study were to investigate the feasibility of improving the efficacy of prevalent models to date (predictive models based on radiomic or deep learning features alone) and to provide a new approach for constructing models for non-invasive detection of EGFR mutations, a and there may be a promise for future extensions to develop models for predicting other genotypes or other tasks.

Tumor heterogeneity (36–38) is the leading driver of drug resistance and disease progression in the post-EGFR-TKI treatment course, and the underlying factor that liquid biopsy and puncture pathology may not reflect the overall truly mutated status of the lesion in the process of disease genetic identification, therapeutic efficacy monitoring and follow-up.

However, Radiogenomics can effectively discern the heterogeneous patterns within tumors through artificial intelligence and mathematical statistics, bridging the limitations of pathological biopsies and liquid biopsies and assisting clinicians in conducting more precise clinical decisions. For remote and impoverished area and countries, this is an inexpensive, low-cost and efficient genetic diagnostic weapon if the radiogenomic model can be brought to clinical practice successfully by future.

The results of this study confirmed the reliability of radiomic and deep learning models for the non-invasive prediction of EGFR mutation status in lung adenocarcinoma with a high degree of accuracy. In lung adenocarcinoma patients, two previous studies (39, 40)combined both radiomic and clinical features to successfully build a radiomic-clinical model that could efficiently identify EGFR mutant phenotypes from wild types with good AUCs of 0.779 and 0.823. However, two other studies (41, 42) also successfully built a combined radiomic-clinical prediction model but also found that a deep learning feature-based model could also predict EGFR gene mutation status in patients with lung adenocarcinoma in a more accurate manner, achieving AUCs of 0.810 and 0.758. These previous findings are consistent with the results of our current study. However, our present differs from these previous studies in that they predominantly applied radiomic and deep learning features separately to build radiomic-clinical models or deep learning models. In this study, we innovatively developed a fusion prediction model to diagnose EGFR mutations in patients with non-small cell lung cancer by fusing the most widely accepted clinicopathological, radiology, and radiomic features with deep learning features. A previous study published findings for a fusion model that were similar to our present results; the efficacy of this previous fusion model was also more efficient than the radiomic model (AUC: 0.831 *vs* 0.758) (25). Comparing to this study, which enrolled only solid lung adenocarcinoma cases, had incomplete coverage of the mutant site, and used thick layers of images, our study also included a significant number of ground glass type non-small cell lung cancer cases and new radiology features. All of the images used in the present study had a layer thickness of <1.5 mm, thus making our models more realistic to the actual clinical scenario, thus providing more applicable data that could support the wider use of these models clinically.

Our current findings confirm the concept of fusing multiple features to build prediction models to enhance the efficacy of individual models. We prove that this strategy is feasible and may be applied to the prediction of other genetic targets in the future, and even to other fields, including the identification of benign and malignant nodes, prediction of the degree of infiltration, as well as the prognosis of survival analysis.

Both Clinicopathology features that gender and smoking history, degree of invasion, and morphology features like cancer density and the vacuole sign, were independent predictors for the EGFR mutant phenotype. The present study reconfirmed that EGFR mutant phenotype is more prevalent in women and non-smoking patients (43–45). In addition, the tumor invasion degree and density are highly associated with the EGFR mutation status. The higher the degree of tumor infiltration and density, the more likely the mutation of EGFR will occur. A greater degree of invasion indicates more heterogeneous cells, faster gene duplication and an increased mutation frequency. This is in line with prior research (46) study 1 where the mutation frequency of EGFR was observed to be much larger and more distinct in IAC, than in MIA, AIS, and AAH. Compared to pure ground glass nodules, mixed ground glass nodules and solid nodules with greater density had significantly better EGFR mutation rates, which also is aligned with previous studies (46, 47) posting that the solid component is remarkably sensitive for diagnosing invasiveness and has a superior EGFR mutation profile. Both vacuole sign and age were correlated with EGFR mutation condition, yet unfortunately, this discovery was not in accordance with the results of earlier studies (48–50), probably because our research center specializes in geriatrics, so the population enrolled is mostly elderly, so there might be a sample error, while the studies correlating vacuole sign and EGFR are fewer, both of which have to be further verified by subsequent research.

Two previous studies incorporated two EGFR-related predictors, gender and smoking history, into the construction of a fused clinical-radiomic model; however, the efficacy of the final separate radiomic model was not improved (51, 52). We also found that several radiology features were not significantly correlated with EGFR mutations, including air bronchogram sign, spiculation sign, and lobulation sign. The involvement of relevant features in model construction did not effectively augment the efficacy of the radiomic model. These highly subjective and time-consuming features should be considered carefully in future studies; deletion of these features may help to streamline the development procedures of radiogenomic predictive models.

Some limitations need to be considered. First, this was a retrospective study. Firstly, EGFR frequently merges with tumor suppressor genes mutations (53), like TP53 (incidence >5%), but in the clinical setting, tumor suppressor genes testing is not routinely conducted, thus the genetic data in this study only contains EGFR synapses, and there is no investigation yet to elucidate whether the effect of the remaining co-alteration mutations upon the radiogenomic model, so more information should be collected on combined mutations for rigorous prospective trials in the future. Second, EGFR mutation prevalence is varying across ethnics, such that it is generally of a higher rate for the Asian population than that of the American and European ones (54), hence the model may be more generalizable to Asia; also, there are large regional diversity in lifestyle practices, which may sometimes change the structuring composition of the model, such as clinical features smoking history. This is why in coming future, multi-center, multi-ethnic studies are expected to validate the robustness and generalization power of radiogenomics models. And lastly: in this study, a time-consuming manual segmentation pattern was implemented; the future semi-automatic or fully automatic segmentation mode with deep learning algorithms should be applied to streamline the whole process.

## Conclusion

Both radiomic models and deep learning models can predict EGFR gene mutation status relatively efficiently and non-invasively. By integrating radiomics and deep learning features, it is possible to build prediction models that can significantly upgrade the performance of the basic radiomic models and help to improve the performance of deep learning models. Models featuring deep learning techniques have the potential for broader application in the non-invasive diagnosis of lung cancer genes mutation.

## Data Availability Statement

The original contributions presented in the study are included in the article/**Supplementary Material**. Further inquiries can be directed to the corresponding author.

## Ethics Statement

This study involved human participants and was reviewed and approved by the Ethics Committee of the Huadong Hospital Affiliated with Fudan University (number:2019K134). Written informed consent for participation was not required for this study in accordance with the national legislation and the institutional requirements.

## Author Contributions

ML and YS conceived the concept of this study. YY and KW collected the data. PG, MT, WM, LQ, JL, WC, LJ, KK, and SD analyzed the data. XH and YS drafted and revised the manuscript. All authors reviewed the manuscript, and ML made corrections to the manuscript. All authors contributed to the article and approved the submitted version.

## Funding

This study was supported by the National Nature Science Foundation of China (Reference: 61976238; ML), and the Shanghai “Rising Stars of Medical Talent” and Youth Development Program “Outstanding Youth Medical Talents” (Reference: SHWSRS(2021)_99), and Scientific Research Program of Shanghai Science and Technology Commission (Reference: 20Y11902900).

## Conflict of Interest

Author KK was employed by Dianei Technology. Author SD was employed by GE Healthcare.

The remaining authors declare that the research was conducted in the absence of any commercial or financial relationships that could be construed as a potential conflict of interest.

## Publisher’s Note

All claims expressed in this article are solely those of the authors and do not necessarily represent those of their affiliated organizations, or those of the publisher, the editors and the reviewers. Any product that may be evaluated in this article, or claim that may be made by its manufacturer, is not guaranteed or endorsed by the publisher.

## Abbreviations

NSCLC, Non-Small Cell Lung Cancer; *EGFR*, Epidermal Growth Factor Receptor; TKI, Tyrosine Kinase Inhibitors; PFS, Progression-Free Survival; OS, Overall Survival; CT, Computed Tomography; MM, Millimeter; WHO, World Health Organization; PCR, Polymerase Chain Reaction; DICOM, Digital Imaging Communications in Medicine; VOI, Volume Region of Interest; ICC, Intraclass Correlation Coefficient; Light GBM, Light Gradient Boosting Machine; HU, Hounsfield Unit; Res-Net, Residual Network; ROC, Receiver Operating Characteristic Curve; PRC, Precision Recall Curve; AUC, Area Under the Curve; GLCM, Gray-Level Co-occurrence Matrix; *ALK*, Anaplastic Lymphoma Kinase; *KRAS*, Kirsten Rat Sarcoma Viral Oncogene Homolog.
