# Supplementary material for: Three-Dimensional Convolutional Neural Network-Based Prediction of Epidermal Growth Factor Receptor Expression Status in Patients With Non-Small Cell Lung Cancer
Source: Front Oncol. 2022 Feb 2;12:772770. doi: 10.3389/fonc.2022.772770 (PMC8848731; doi:10.3389/fonc.2022.772770)
Supplement: Supplementary file 1 [file DataSheet_1.docx]

**Supplementary Table 1:** The parameters of scanning instruments.

|  | GE Discovery  CT750 HD | LightSpeed  VCT | Somatom  Sensation 16 |
| --- | --- | --- | --- |
| Tube voltage | 120 kV | 120 kV | 120 kV |
| Tube current | 200 mA | 200 mA | 200 mA |
| Pitch | 0.984:1 | 0.984:1 | 0.8 |
| Collimation | 0.625 mm × 64 | 0.625 mm × 64 | 0.75 mm × 16 |
| SFOV | 50 cm | 50 cm | 50 cm |
| Slice thickness of reconstruction | 1.25 mm | 1.25 mm | 1/1.5 mm |
| Reconstruction algorithm | STND | STND | Medium sharp |
| Distribution in the training set | 340 | 184 | 246 |
| Distribution in the test set | 135 | 83 | 86 |

**Supplementary Table 2:** The distribution of clinical-radiology features between the training and test set.

| **Characteristics** | **Train** | **Test** | ***p-value*** |
| --- | --- | --- | --- |
| **Genetic type** |  |  | ***0.982*** |
| Wild type | 378(49.1) | 149(49.0) |  |
| Mutation | 392(50.9) | 155(51.0) |  |
| **Gender** |  |  | ***0.957*** |
| Male | 318(41.3) | 125(41.1) |  |
| Female | 452(58.7) | 179(58.9) |  |
| **Age** | 59.0(19.0) | 59.0(19.0) | ***0.478*** |
| **Invasive Degree** |  |  | ***0.595*** |
| Non-invasive | 91(11.8) | 38(12.5) |  |
| Micro-invasive | 289(37.5) | 104(34.2) |  |
| Invasive | 390(50.6) | 162(53.3) |  |
| **Location** |  |  | ***0.709*** |
| RUL | 282(36.6) | 104(34.2) |  |
| RML | 62(8.1) | 28(9.2) |  |
| RLL | 123(16.0) | 58(19.1) |  |
| LUL | 201(26.1) | 76(25.0) |  |
| LLL | 102(13.2) | 38(12.5) |  |
| **Cancer density** |  |  | ***0.272*** |
| Pure GGO | 101(13.1) | 40(13.2) |  |
| Mixed GGO | 534(69.4) | 198(65.1) |  |
| Solid | 135(17.5) | 66(21.7) |  |
| **Border** |  |  | ***0.488*** |
| Well-define | 525(68.2) | 209(68.8) |  |
| Less-define | 140(18.2) | 61(20.1) |  |
| Ill-define | 105(13.6) | 34(11.2) |  |
| **Vacuolation** |  |  | ***0.552*** |
| Present | 314(40.8) | 130(42.8) |  |
| Absent | 456(59.2) | 174(57.2) |  |
| **Air Bronchogram** |  |  | ***0.691*** |
| Present | 314(40.8) | 128(42.1) |  |
| Absent | 456(59.2) | 176(57.9) |  |
| **Spiculation** |  |  | ***0.291*** |
| Short | 176(22.9) | 76(25.0) |  |
| Deep | 58(7.5) | 24(7.9) |  |
| Mixed | 141(18.3) | 67(22.0) |  |
| Absent | 395(51.3) | 137(45.1) |  |
| **Lobulation** |  |  | ***0.140*** |
| Shallow | 239(31.0) | 77(25.3) |  |
| Deep | 15(1.9) | 7(2.3) |  |
| Mixed | 511(66.4) | 215(70.7) |  |
| Absent | 5(0.6) | 5(1.6) |  |
| **Halo** |  |  | ***0.487*** |
| Present | 122(15.8) | 43(14.1) |  |
| Absent | 648(84.2) | 261(85.9) |  |
| **Vascular- Alteration** |  |  | ***0.623*** |
| Present | 395(51.3) | 161(53.0) |  |
| Absent | 375(48.7) | 143(47.0) |  |
| **Pleural- Indentation** |  |  | ***0.895*** |
| Present | 320(41.6) | 125(41.1) |  |
| Absent | 450(58.4) | 179(58.9) |  |
| **Umbilicated- Indentation** |  |  | ***0.732*** |
| Present | 88(11.4) | 37(12.2) |  |
| Absent | 682(88.6) | 267(87.8) |  |
| **Smoke History** |  |  | ***0.734*** |
| Yes | 318(41.3) | 129(42.4) |  |
| No | 452(58.7) | 175(57.6) |  |

**Note:** RUL: right upper lobe. RML: right middle lobe. RLL: right lower lobe. LUL: left upper lobe. LLL: left lower lobe. GGO: ground glass opacity. Categorical variables (e.g. gender) are expressed by a **number (percentage)**, continuous variables (e.g. age) are expressed by the Median (interquartile range). *p<0.05 (significant), P-values taken with three decimal places equal to 0.000 are expressed as <0.001.

**Supplementary Table 3:** The top 20/108 radiomics features (the highest mutual information) after secondary screening.

| **The top 20 radiomic features** |
| --- |
| wavelet-HLH_ gldm_ Large Dependence High Gray Level Emphasis  log-sigma-1-0-mm-3D_gldm_LowGrayLevelEmphasis  wavelet-HLL_ glrlm Gray Level Variance  log-sigma-4-0-mm-3D_gldm_HighGrayLevelEmphasis  wavelet-HHH_glcm_Imc1  log-sigma-5-0-mm-3D_glrlm_HighGrayLevelRunEmphasis  wavelet-LLL_ glcm_ Difference Entropy  log-sigma-1-0-mm-3D_glszm_GrayLevelNonUniformityNormalized  wavelet-HLH_ glszm_ Zone Percentage  log-sigma-4-0-mm-3D_glrlm_RunPercentage  wavelet-LHL_ first order_ Uniformity  wavelet-HHL_ glszm_ High Gray Level Zone Emphasis  wavelet-LHH_ glrlm_ Short Run Emphasis  wavelet-LLL_ glcm_ Sum Entropy  original_shape_Maximum2DDiameterColumn  original_ first order_ Energy  original_ first order_ Total Energy  wavelet-HLH_ glrlm_ Run Variance  log-sigma-4-0-mm-3D_firstorder_90Percentile  original_ glcm_ Cluster Prominence |

**Note:** For a detailed definition of the categories mentioned above, refer to the pyradiomics website.

**Supplementary Table 4:** Evaluation of performance variation across models ( De-long test)

| **De-long test** | | | | | |
| --- | --- | --- | --- | --- | --- |
|  | **Model**  **_clinical_** | **Model**  **_radiomic_** | **Model**  **_radiomic+clinical_** | **Model**  **_CNN_** | **Model**  **_CNN+radiomic+clinical_** |
| **Model**  **_clinical_** | *-* | *0.596* | *0.491* | *0.038** | *0.000** |
| **Model**  **_radiomic_** | *-* | *-* | *0.876* | *0.072* | *0.006** |
| **Model**  **_radiomic+clinical_** | *-* | *-* | *-* | *0.108* | *0.007** |
| **Model**  **_CNN_** | *-* | *-* | *-* | *-* | *0.580* |
| **Model**  **_CNN+radiomic+clinical_** | *-* | *-* | *-* | *-* | *-* |

**Note：**The numbers given in the table represent p-values; p < 0.05 was considered statistically significant. *indicates a significant difference between models.
